# Hypertension, dietary fiber intake, and cognitive function in older adults [from the National Health and Nutrition Examination Survey Data (2011–2014)]

**DOI:** 10.3389/fnut.2022.1024627

**Published:** 2022-10-21

**Authors:** HuanRui Zhang, Wen Tian, GuoXian Qi, YuJiao Sun

**Affiliations:** Department of Geriatric, The First Hospital of China Medical University, Shenyang, China

**Keywords:** dietary fiber, hypertension, cognitive function, older adults, NHANES

## Abstract

**Background:**

Dietary fiber was associated with hypertension (HYP) and cognitive function, but it was unknown whether the effect of HYP on cognitive function in older adults was modified by dietary fiber intake.

**Methods:**

We recruited 2,478 participants from the 2011–2012 and 2013–2014 National Health and Nutrition Examination Survey (NHANES), with cognitive performance measured by Registry for Alzheimer's disease (CERAD), the Animal Fluency test (AFT), and the Digit Symbol Substitution test (DSST). Multivariate General linear model was used to estimate the interaction between dietary fiber intake and HYP status in association with low cognitive performance.

**Results:**

Among 2,478 participants, 36% was Controlled HYP, 25% was Low uncontrolled HYP, 11% was High uncontrolled HYP, and 86% was low dietary fiber intake. The association between HYP status and DSST impairment differed by dietary fiber intake for those with high uncontrolled HYP compared to those without HYP. Among participants with low dietary fiber intake, those with uncontrolled HYP had higher risk of DSST impairment compared to those without HYP [HYP ≥ 90/140: OR (95% CI), 1.68 (1.15–2.45); HYP ≥ 100/160: OR (95%CI), 2.05 (1.29–3.23)]; however, there was no association between HYP status and DSST impairment among participants with high dietary fiber intake. Moreover, the interaction of HYP status and dietary fiber intake on DSST was close to statistical significance (*P* for interaction = 0.057).

**Conclusions:**

Uncontrolled HYP was associated with poorer cognitive performance in older adults with low, but not high dietary fiber intake. Sufficient dietary fiber intake might be as a new nutrition strategy for the prevention of cognitive impairment in older adults with uncontrolled HYP.

## Introduction

With aging aggravated, the prevalence of age-related cognitive decline increases significantly. The global prevalence of dementia is estimated to reach 131.5 million in 2050, doubled every 20 years (1). The economic damage caused by dementia is about $81 billion (USD) every year, and projected to $2 trillion by 2030 (1). Mild cognitive impairment (MCI) is considered to the preclinical stage of dementia and Alzheimer's disease (AD), the maintenance of good cognitive function and early detection of MCI will help to greatly reduce the burden of public health-care, morbidity and mortality (2). Hypertension (HYP) has long been regarded as the leading cause of age-related cognitive impairment (3). HYP was strongly related to poor cognitive function (4), MCI (5) and dementia (6, 7). Elevated blood pressure accelerated progression and worsening of cognition in people suffering from MCI (8). However, the association of antihypertensive therapy with cognitive impairment still remains controversial (9–12). Therefore, how to improve the cognitive function of older adults with HYP is still facing great challenge. Modifiable lifestyle factors as the important candidate therapeutics are increasingly involved in prevention and treatment of cognitive impairment (13). It is necessary to identify potential modifiable lifestyle factors of cognitive impairment, which will be helpful to prevent and delay cognitive impairment.

Dietary nutrients are regarded as the main modifiable lifestyle factors of many chronic disease, a major nutrient of which is dietary fiber (13–15). Dietary fiber is widely accepted as a healthful nutrient that originate from plant foods (16). Dietary fiber is closely associated with reduced risks of type 2 diabetes (17), stroke (18), cardiovascular disease, cancer (19) and mortality (20). Emerging epidemiologic evidences suggested dietary fiber was associated with cognitive function, high dietary fiber intake reduced the risk of cognitive decline and dementia (21, 22). In a mouse model, the chronic dietary fiber deficiency caused cognitive impairment through gut-brain axis (23). However, it is still unknown whether dietary fiber could influence the relationship between HYP and cognitive function. Given the protective role of dietary fiber for cognitive function, the study is to assess whether the association between HYP and cognitive function differs by dietary fiber intake in older adults, based on the National Health and Nutrition Examination Survey (NHANES) database in the US.

## Methods

### Data source and participants

For the present study, we analyzed secondary data from the 2011–2012 and 2013–2014 NHANES. The NHANES is a stratified, multistage, cross-sectional survey of the U.S. civilian non-institutionalized population conducted by the National Center for Health Statistics (NCHS). NCHS Ethics Review Board approved this protocol, and all participants provided informed consent forms. In this study, we recruited 2,934 participants aged 60 years and older with complete cognitive function assessment test. Additionally, we excluded participants with incomplete dietary fiber intake data (*N* = 221), blood pressure measurement data (*N* = 116), and other potential confounding (*N* = 257). Finally, a total of 2,478 participants were included in the subsequent analysis.

### Cognitive function assessment

Cognitive function was measured by a series of assessments in NHANES 2011–2014, including word learning and recall modules from the Consortium to Establish a Registry for Alzheimer's disease (CERAD), the Animal Fluency test (AFT), and the Digit Symbol Substitution test (DSST).

The CERAD word learning and recall modules were applied to assess immediate and delayed learning ability for new verbal information (24). The CERAD test consists of three immediate recalls (CERAD-WL), and a delayed recall (CERAD-DR). For the immediate recalls, participants were instructed to read 10 unrelated words, and they were asked to recall as many words as possible immediately. The delayed recall occurred after the AFT and DSST were completed. The AFT was used to examine verbal fluency (25). Participants were required to recall as many animals as possible in 1 min. The DSST was applied to assess the abilities of processing speed, sustained attention, and working memory (26). Participants were asked to copy the corresponding symbols in the 133 boxes in 2 min.

As there is no recommended standard for cognitive impairment by CERAD-WL, CERAD-DR, AFT and DSST, the lowest quartile of these four scores was used as the cut-off points, which were adjusted according to age (Table 1) (27). Participants with scores lower than or equal to the cut-off points were defined as cognitive impairment.

**Table 1:** The cutoff points of cognitive function test scores adjusted by age.

**Cognitive test**	**Cut off points of test**		
	**≥60 years**	**≥70 years**	**≥80 years**
Immediate recall (CERAD-WL) score	17	16	14
Delayed recall (CERAD-DR) score	5	4	3
Verbal fluency (AFT) score	14	12	12
Executive function & processing speed (DSST) score	37	33	29

### Dietary fiber intake

The independent variable was dietary fiber intake (g), which was obtained from the 24-h recall survey. Dietary fiber intake was calculated according to the US Department of Agriculture (USDA) Food and Nutrient Databases for Dietary Studies (FNDDS). The first 24-h recall survey was conducted in the Mobile Examination Center (MEC) and the second was collected by telephone 3–10 days later. The dietary fiber intake was calculated as an average of 2 days dietary recall data if 2 days data was available. Otherwise, single dietary recall was used. We grouped the dietary fiber intake into high level (>25 g/day) and low level (≤25 g/day), which is consistent with the American Heart Association recommendations for dietary fiber intake of at least 25 g/day for adults (28, 29).

### HYP status

Blood pressure measurement data were measured by trained interviewers in the MEC. We defined four categories of HYP status according to their answers and the results of BP measurements: NO HYP [no self-reported hypertension, no self-reported use of anti-hypertensive medications, and systolic blood pressure (SBP) <140 mmHg or/and diastolic blood pressure (DBP) <90 mmHg]; Controlled HYP (self-reported hypertension, self-reported use of anti-hypertensive medications, and SBP <140 mmHg or/and DBP <90 mmHg); Uncontrolled HYP (included untreated HYP and treated but uncontrolled HYP) was divided into two groups by BP status: 1. Low uncontrolled HYP: BP ≥140/90 mmHg (140 ≤ SBP <160 mmHg or/and 90 ≤ DBP <100 mmHg), 2. High uncontrolled HYP: BP ≥160/100 mmHg (SBP ≥160 mmHg or/and DBP ≥100 mmHg).

### Covariates

The sociodemographic information, lifestyle factors and medical-related information were recorded. The sociodemographic information included age, sex, ethnicity (non-Hispanic White, non-Hispanic Black, other Hispanic, and other race), marital status (married/with a partner, unmarried and other), education (less than 11th grade and high-school grade and above). The lifestyle factors included smoking (non-smoker, former smoker, and current smoker) and sport (≤150 min/week, and more than 150 min/week). The total amount of sport was assessed by summing both moderate- and vigorous-intensity levels activity (weekly occupational, recreational, and transportation physical) multiplied by the number of days by minutes per day (frequency × duration). The medical-related information included body mass index (BMI), total cholesterol (TC, mg/dL), high density lipoprotein cholesterol (HDL-C, mg/dL), diabetes mellitus, cardiovascular disease (CVD), and treatments (antihypertensive drugs, hypoglycemic agents, lipid-lowering drugs, antiplatelet drugs). The lipid profile level was presented as a ratio of TC to HDL-C. Diabetes mellitus was defined as self-reported diabetes, hemoglobin A1c ≥6.5%, fasting plasma glucose level ≥126 mg/dl, or reported use of oral glucose-lowering medication or insulin. CVD was defined as self-reported diagnosis of heart failure, coronary heart disease, angina, heart attack or stroke.

### Statistical analysis

Characteristics of participants aged 60 years and older were summarized by HYP status (No HYP; Controlled HYP; Low uncontrolled HYP; High uncontrolled HYP). We presented means and standard deviation for continuous variables, and counts and proportions for categorical variables. Continuous variables and categorical variables were compared using ANOVA and Pearson's χ^2^ test, separately. The odds ratio (OR) with 95% confidence interval (CI) for HYP status were evaluated using multivariate General linear model (GLM), using the No HYP as the reference. Trend test was used to check the change of cognition in different HYP status. Our primary analyses proceeded in three stages. First, we explored the associations between the HYP status with cognitive impairment among all participants. Second, to test whether the association differed between high and low dietary fiber intake level, the study sample was divided into high- and low-dietary fiber intake subgroups, then the relationship was estimated in different subgroups. Third, the interaction model by HYP status and dietary fiber intake was constructed to study whether the interaction existed. Models were adjusted by potential covariates, including age, gender, ethnicity, marital status, education, smoking, BMI, smoking, sport, TC/HDL-C, diabetes, CVD, antihypertensive drugs, hypoglycemic agents, lipid-lowering drugs, antiplatelet drugs. R version 4.0.3 was used for analyses, and a two-sided *p*-value < 0.05 was considered significant.

## Results

### Baseline characteristics of participants

A total of 2,478 participants aged 60 years or older from NHANES 2011–2014 were included in this study. The basic characteristics of participants by HYP status are shown in Table 2. The mean (SE) age was 69.36 ± 6.76 years and 50.2% of participants was women. Participants with high uncontrolled HYP were more likely to had less dietary fiber intake and sport, be older, female, Non-Hispanic Black, less educated, more fat, current smoker, unhealthier in terms of history of diabetes and CVD (all *P* < 0.05). And treatments, including antihypertensive drugs, hypoglycemic agents, lipid-lowering drugs, antiplatelet drugs, are also shown in Table 2.

**Table 2:** Characteristics of participants aged 60 years and older from the National Health and Nutrition Examination Survey (NHANES) (2011–2014).

**Characteristics**	**NO HYP** **(*N* = 698)**	**Controlled HYP** **(*N* = 890)**	**Low uncontrolled HYP (*N* = 629)**	**High uncontrolled HYP (*N* = 261)**	***P*** **-value**
Age, years	68.11 ± 6.48	69.22 ± 6.64	69.97 ± 6.77	71.73 ± 7.07	<0.001
Sex-female, *n* (%)	325 (46.6)	458 (51.5)	314 (49.9)	147 (56.3)	0.042
Ethnicity, *n* (%)					<0.001
Non-Hispanic White	385 (55.2)	453 (50.9)	285 (45.3)	117 (44.8)	
Non-Hispanic Black	93 (13.3)	231 (26.0)	162 (25.8)	76 (29.1)	
Mexican American/Hispanic	82 (11.7)	78 (8.8)	70 (11.1)	24 (9.2)	
Other	138 (19.8)	128 (14.4)	112 (17.8)	44 (16.9)	
Education-higher than high school, *n* (%)	551 (78.9)	664 (74.6)	471 (74.9)	185 (70.9)	0.046
Marital status-married, *n* (%)	403 (57.7)	506 (56.9)	339 (53.9)	135 (51.7)	0.246
Body mass index, kg/m^2^	27.72 ± 5.74	30.65 ± 6.48	28.89 ± 6.35	27.87 ± 5.67	<0.001
Smoking, *n* (%)					0.041
Never	342 (49.0)	421 (47.3)	310 (49.3)	132 (50.6)	
Former	266 (38.1)	373 (41.9)	221 (35.1)	91 (34.9)	
Current	90 (12.9)	96 (10.8)	98 (15.6)	38 (14.6)	
Dietary fiber, g/day	18.07 ± 10.19	16.39 ± 8.41	16.96 ± 8.97	15.90 ± 8.40	0.001
Sport, *n* (%)					<0.001
Low > 150	312 (44.7)	502 (56.4)	354 (56.3)	142 (54.4)	
High ≤ 150	386 (55.3)	388 (43.6)	275 (43.7)	119 (45.6)	
Total cholesterol /High density lipoprotein cholesterol	3.79 ± 1.21	3.69 ± 1.14	3.79 ± 1.26	3.86 ± 1.35	0.120
Diabetes diagnosis, *n* (%)					<0.001
Absence	563 (80.7)	575 (64.6)	443 (70.4)	167 (64.0)	
Presence	135 (19.3)	315 (35.4)	186 (29.6)	94 (36.0)	
Cardiovascular disease diagnosis, *n* (%)					
Absence	607 (87.0)	635 (71.3)	498 (79.2)	196 (75.1)	<0.001
Presence	91 (13.0)	255 (28.7)	131 (20.8)	65 (24.9)	
Antihypertensive drugs, *n* (%)					
Absence	698 (100.0)	79 (8.9)	256 (40.7)	88 (33.7)	<0.001
Presence	0 (0.0)	811 (91.1)	373 (59.3)	173 (66.3)	
Hypoglycemic agents, *n* (%)					
Absence	605 (86.7)	652 (73.3)	496 (78.9)	198 (75.9)	<0.001
Presence	93 (13.3)	238 (26.7)	133 (21.1)	63 (24.1)	
Lipid-lowering drugs, *n* (%)					
Absence	495 (70.9)	396 (44.5)	368 (58.5)	164 (62.8)	<0.001
Presence	203 (29.1)	494 (55.5)	261 (41.5)	97 (37.2)	
Antiplatelet drugs, *n* (%)					
Absence	676 (96.8)	807 (90.7)	594 (94.4)	243 (93.1)	<0.001
Presence	22 (3.2)	83 (9.3)	35 (5.6)	18 (6.9)	

### Cognitive function of participants

The four cognitive function tests were all associated with HYP status (Table 3). Participants with high uncontrolled HYP tended to have lower CERAD-WL, CERAD-DR, AFT and DSST (all *P* < 0.001). Cognitive impairment was defined according to the cutoff points of cognitive function test scores adjusted by age (Table 1). Participants with high uncontrolled HYP were more likely to be AFT impairment (*P* = 0.001), and DSST impairment (*P* < 0.001).

**Table 3:** The cognitive function of participants aged 60 years and older from the National Health and Nutrition Examination Survey (NHANES) (2011–2014).

**Cognitive test**	**NO HYP** **(*N* = 698)**	**Controlled HYP** **(*N* = 890)**	**Low uncontrolled HYP (*N* = 629)**	**High uncontrolled HYP (*N* = 261)**	**P value**
Immediate Recall (CERAD-WL) score	19.67 ± 4.42	19.13 ± 4.46	18.65 ± 4.72	18.17 ± 4.61	<0.001
Delayed Recall (CERAD-DR) score	6.27 ± 2.28	6.05 ± 2.26	5.78 ± 2.36	5.62 ± 2.20	<0.001
Verbal Fluency (AFT) score	17.97 ± 5.73	16.63 ± 5.32	16.37 ± 5.21	15.41 ± 5.28	<0.001
Executive function & processing speed (DSST) score	50.44 ± 17.19	46.83 ± 16.69	44.58 ± 16.78	41.00 ± 16.43	<0.001
Immediate Recall (CERAD-WL) impairment, *n* (%)^§^					0.277
Absence	508 (72.8)	644 (72.4)	435 (69.2)	178 (68.2)	
Presence	190 (27.2)	246 (27.6)	194 (30.8)	83 (31.8)	
Delayed Recall (CERAD-DR) impairment, *n* (%)^§^					0.509
Absence	495 (70.9)	647 (72.7)	440 (70.0)	179 (68.6)	
Presence	203 (29.1)	243 (27.3)	189 (30.0)	82 (31.4)	
Verbal Fluency (AFT) impairment, *n* (%)^§^					0.001
Absence	534 (76.5)	615 (69.1)	442 (70.3)	170 (65.1)	
Presence	164 (23.5)	275 (30.9)	187 (29.7)	91 (34.9)	
Executive function & processing speed (DSST) impairment, *n* (%)^§^					<0.001
Absence	561 (80.4)	664 (74.6)	442 (70.3)	169 (64.8)	
Presence	137 (19.6)	226 (25.4)	187 (29.7)	92 (35.2)	

§ The cognitive impairment was defined according to the cutoff points of cognitive function test scores adjusted by age (Table 1).

### Association between HYP status and cognitive impairment

While comparing to the NO HYP group, the multivariate adjusted GLM indicated that participants with high uncontrolled HYP was associated with 56% elevated risk of DSST impairment [OR (95% CI), 1.56 (1.09–2.21)], and participants with high uncontrolled HYP was associated with 87% elevated risk of DSST impairment [OR (95% CI), 1.87 (1.21–2.88)] (Table 4). However, there was no association with CERAD or AFT impairment for those with uncontrolled HYP compared to those without HYP.

**Table 4:** HYP status and cognitive function impairment by levels of fiber intake among participants aged 60 years and older from the National Health and Nutrition Examination Survey (NHANES) (2011–2014).

**Characteristics**	**Overall** ^§^		**Low fiber intake (≤ 25 g/day)** ^§^		**High fiber intake (>25 g/day)** ^§^		**P for interaction**
	**OR (95% CI)**	***P*** **-value**	**OR (95% CI)**	***P*** **-value**	**OR (95% CI)**	***P*** **-value**	
Immediate Recall (CERAD-WL)							0.941
NO HYP	1 (Ref)		1 (Ref)		1 (Ref)		
Controlled HYP	1.22 (0.88–1.71)	0.235	1.18 (0.82–1.69)	0.381	1.64 (0.65–4.17)	0.294	
Low uncontrolled HYP	1.24 (0.93–1.67)	0.145	1.23 (0.89–1.69)	0.208	1.58 (0.72–3.47)	0.253	
High uncontrolled HYP	1.26 (0.86–1.82)	0.232	1.26 (0.84–1.87)	0.265	1.34 (0.40–4.20)	0.624	
*P* for trend		0.208		0.215		0.449	
Delayed Recall (CERAD-DR)							0.171
NO HYP	1 (Ref)		1 (Ref)		1 (Ref)		
Controlled HYP	1.02 (0.73–1.42)	0.917	1.04 (0.72–1.48)	0.848	1.02 (0.41–2.51)	0.971	
Low uncontrolled HYP	1.05 (0.78–1.40)	0.748	1.08 (0.78–1.49)	0.633	0.94 (0.43–1.99)	0.867	
High uncontrolled HYP	1.11 (0.77–1.61)	0.569	1.17 (0.79–1.74)	0.431	0.81 (0.22–2.58)	0.736	
*P* for trend		0.527		0.379		0.713	
Verbal Fluency (AFT)							0.075
NO HYP	1 (Ref)		1 (Ref)		1 (Ref)		
Controlled HYP	1.21 (0.86–1.71)	0.268	1.19 (0.82–1.72)	0.359	1.60 (0.59–4.24)	0.347	
Low uncontrolled HYP	1.07 (0.78–1.45)	0.681	1.15 (0.83–1.60)	0.405	0.65 (0.26–1.56)	0.346	
High uncontrolled HYP	1.20 (0.82–1.76)	0.341	1.27 (0.84–1.91)	0.248	0.60 (0.15–2.00)	0.426	
*P* for trend		0.747		0.351		0.070	
Executive function & processing speed (DSST)							0.057
NO HYP	1 (Ref)		1 (Ref)		1 (Ref)		
Controlled HYP	1.41 (0.95–2.10)	0.090	1.44 (0.94–2.21)	0.093	1.43 (0.43–4.67)	0.557	
Low uncontrolled HYP	**1.56 (1.09–2.21)**	**0.014**	**1.68 (1.15–2.45)**	**0.008**	1.55 (0.54–4.40)	0.412	
High uncontrolled HYP	**1.87 (1.21–2.88)**	**0.005**	**2.05 (1.29–3.23)**	**0.002**	0.89 (0.18–3.94)	0.878	
*P* for trend		**0.003**		**0.001**		0.816	

§ GLM model were adjusted by age, gender, ethnicity, marital status, education, smoking, body mass index, smoking, sport, total cholesterol/high density lipoprotein cholesterol, diabetes, cardiovascular disease, antihypertensive drugs, hypoglycemic agents, lipid-lowering drugs, antiplatelet drugs.

The bold values indicate the significant difference.

### Association between HYP status and cognitive impairment differed by dietary fiber intake

The association between HYP status and DSST impairment differed by dietary fiber intake for those with high uncontrolled HYP compared to those without HYP (*P* for interaction = 0.057), though the interaction of HYP status and dietary fiber intake was close to statistically significant (Table 4). Among participants with low dietary fiber intake, those with uncontrolled HYP had higher risk of DSST impairment compared to those without HYP [HYP ≥ 90/140: OR (95% CI), 1.68 (1.15–2.45); HYP ≥ 100/160: OR (95% CI), 2.05 (1.29–3.23)]; however, there was no association between HYP status and DSST impairment among participants with high dietary fiber intake (Table 4).

## Discussion

The study, a nationally representative sample of older adults in the US based on NHANES 2011–2014, focused on whether the association of HYP with cognitive function was affected by dietary fiber intake. The results found that older adults with uncontrolled HYP were significantly associated with poorer executive function and processing speed (assessed by DSST score), compared to those without HYP. No significant associations were found for cognitive performance in immediate recall, delayed recall and verbal fluency. Notably, the associations of HYP with cognitive performance differed by dietary fiber intake in older adults, compared to those without HYP. In older adults with low dietary fiber intake, those with uncontrolled HYP were associated with poorer executive function and processing speed compared to those without HYP; but in those with high dietary fiber intake, no differences were found. The higher the blood pressure, the poorer executive function and processing speed. But the similar trend was not found in those with high dietary fiber intake. And further analysis revealed the interactive impact of HYP status and dietary fiber intake on DSST was close to statistical significance. The study suggested high dietary fiber intake might have contributed to regulate the adverse effect of uncontrolled HYP on cognitive impairment in older adults.

Some prospective cohort studies confirmed the causal association of HYP with CI and AD (30). The Honolulu-Asia Aging Study found the relationship between mid-life higher blood pressure and CI and AD in later life (31). Among participants with HYP (SBP ≥160 mmHg) had a 4.8-fold increased risk of dementia, compared to those without HYP (31). The prospective studies in Finland and US found similar results (32, 33). Evidences have indicated that HYP impacts various fields of cognitive function (30). HYP was negatively associated with cognitive function, including abstract reasoning, executive function, processing speed and memory (34). The DSST is regarded as a more sensitive assessment of cognitive impairment than the Mini-Mental State Examination (MMSE), which can accurately predict brain dysfunction and identify cognitive impairment in older adults (35). A study found the significant negative relationship between higher BP and poorer cognitive function assessed by DSST in men aged 45–55 years, and higher SBP was associated with a lower DSST score in older women at 8 years of follow-up (36). In agreement with the previous evidences, our results showed that uncontrolled HYP was significantly associated with poorer cognitive performance in executive function and processing speed among older adults.

It was confirmed that high dietary fiber intake reduced the risk of HYP (37, 38). A study from NHANES found a non-linear relationship between dietary fiber and HYP, the risk of HYP decreased by 53% when dietary fiber intake increased from 0.07 to 0.35 g/kg/day (39). Our previous study suggested dietary fiber intake was significantly inversely associated with all-cause and CVD mortality in older adults with HYP (40). A positive relationship between dietary fiber intake and cognitive function in older adults has been reported (41, 42). In a cohort of adults ≥50 years, a dietary fiber-rich diet significantly improved the cognitive function during the period of 10-year follow up (42). The most recent study from NHANES 2011–2014 found dietary fiber was significantly associated with domain-specific cognition (DSST, executive function and processing speed) in older adults ≥60 years, and a linear dose-response relationship between dietary fiber and DSST until a dietary fiber intake of 34 g/d (21). Based on these previous researches, our study extended these findings on the relationship between HYP and cognitive function by estimating how dietary fiber modified their association. Our study suggested that the adverse associations of HYP with executive function and processing speed were alleviated in older adults with uncontrolled HYP for those with high dietary fiber intake, compared to those without HYP. And the interaction between dietary fiber and uncontrolled HYP on DSST impairment was close to statistical significance. The results suggested that the association between HYP and cognitive function was inconsistent at different levels of dietary fiber intake, the adverse relationship between uncontrolled HYP and cognitive function might be modified by dietary fiber intake. Increasing dietary fiber intake may be a potential therapeutic target for cognitive impairment in older adults with uncontrolled HYP.

Chronic, uncontrolled HYP is an important risk factor for ischemic and hemorrhagic stroke, causes a 3–6-fold increased risk of cognitive impairment (43). Studies demonstrated a beneficial effect of dietary fiber intake on stroke risk (44, 45). A meta-analysis of prospective studies suggested that every 10 grams of dietary fiber added to daily diet, the risk of stroke decreased by 12% (46). Dietary fiber exerted a beneficial effect on stroke probably by improving blood lipid profile, glucose, insulin sensitivity, chronic inflammation and fibrinolytic activity (46, 47). The other potential mechanism that the beneficial effect of dietary fiber intake on cognitive function may be due to regulation of gut microbiota on gut-brain axis (48). Dietary fiber is fermented by gut microbiota in the colon, which shapes the gut microbiota and promotes short-chain fatty acids as fermentative end products (49). Dietary fiber modulates and improves cognitive function *via* the gut-brain axis by their fermentative end products (50). For older adults with uncontrolled HYP, dietary fiber may play a more important role in improving cognitive function, compared to these without HYP. But the exact mechanism remains to be uncertain, needs to be further studied. There exists the health benefit of dietary fiber, but in fact the daily intake of dietary fiber is seriously inadequate around the world. The average intake of dietary fiber is about 15 g/day in the US (51), 13.6 g/day in the UK (52), and 11 g/day in China (53), which is significantly lower than the 25–35 g/day of the World Health Organization daily recommended dietary fiber intake. In our study, the average dietary fiber intake was 16.96 g/day, older adults with uncontrolled HYP had significantly lower dietary fiber intake than these without HYP. The results suggested the sufficient intake of dietary fiber might be regarded as a nutritional intervention to reduce cognitive impairment in older adults with uncontrolled HYP.

There are still certain limitations in the study. The cross-sectional study did not deduce the causal relationship between dietary fiber and HYP, cognitive function in older adults. The data of dietary fiber intake was obtained from 24-h dietary recall interviews, which might cause self-reports bias. Three cognitive assessment methods, including the CERAD, AFT and DSST were used in the study, but these tests did not contain all domains of cognitive function, our results might be not suitable for other domains of cognitive function. Many known confounding factors have been adjusted, but there are still other potential unmeasured confounders were not included in the study. The sample size was further reduced in the high dietary fiber intake group, and the larger samples need to be explored in future studies. Despite its limitations, the study explored the potential protective role of high dietary fiber intake in cognitive impairment and the potential interaction with HYP.

## Conclusion

The results found uncontrolled HYP is associated with decreased executive function and processing speed in older adults, high dietary fiber intake might mitigate their negative relationships. It suggested adequate dietary fiber intake might be required to protect against cognitive impairment in older adults with uncontrolled HYP. This study might provide a new nutrition management strategy for the prevention of cognitive impairment in older adults with uncontrolled HYP, health managers should consider nutrition screening to encourage them for increasing dietary fiber intake. The future prospective and intervention studies are needed to confirm our results.

## Data availability statement

Publicly available datasets were analyzed in this study. This data can be found here: https://wwwn.cdc.gov/nchs/nhanes/continuousnhanes/default.aspx?BeginYear=2011 and https://wwwn.cdc.gov/nchs/nhanes/continuousnhanes/default.aspx?BeginYear=2013.

## Ethics statement

The survey protocol was approved by NCHS Ethics Review Board (https://www.cdc.gov/nchs/nhanes/irba98.htm), and all participants have provided written informed consent.

## Author contributions

HZ participated in the design of this study, acquired data, performed the statistical analysis, and drafted the manuscript. WT and GQ participated in its design. YS conceived of the study, participated in its design and drafting, and provided critical revision for important intellectual content. All authors contributed to the article and approved the submitted version.

## Conflict of interest

The authors declare that the research was conducted in the absence of any commercial or financial relationships that could be construed as a potential conflict of interest.

## Publisher's note

All claims expressed in this article are solely those of the authors and do not necessarily represent those of their affiliated organizations, or those of the publisher, the editors and the reviewers. Any product that may be evaluated in this article, or claim that may be made by its manufacturer, is not guaranteed or endorsed by the publisher.
